# The role of kidney injury biomarkers in COVID-19

**DOI:** 10.1080/0886022X.2022.2107544

**Published:** 2022-08-05

**Authors:** Lianjiu Su, Jiahao Zhang, Zhiyong Peng

**Affiliations:** a Department of Critical Care Medicine, Zhongnan Hospital of Wuhan University, Wuhan, China; b Department of Cardiology, David Geffen School of Medicine, University of California, Los Angeles, CA, USA; c Department of Critical Care Medicine, Center of Critical Care Nephrology, University of Pittsburgh Medical Center, Pittsburgh, PA, USA

**Keywords:** Acute kidney injury, biomarker, COVID-19

## Abstract

The coronavirus disease-2019 (COVID-19) outbreak has been declared a global pandemic. COVID-19-associated acute kidney injury (COVID-19 AKI) is related to a high mortality rate and serves as an independent risk factor for hospital death in patients with COVID-19. Early diagnosis would allow for earlier intervention and potentially improve patient outcomes. The goal of early identification of AKI has been the primary impetus for AKI biomarker research, and several kidney injury biomarkers have been demonstrated to be beneficial in predicting COVID-19 AKI as well as disease progression in COVID-19. Furthermore, such data provide valuable insights into the molecular mechanisms underlying this complex and unique disease and serve as a molecular phenotyping tool that could be utilized to direct clinical intervention. This review focuses on a number of kidney injury biomarkers, such as CysC, NAGAL, KIM-1, L-FABP, IL-18, suPAR, and [TIMP-2] • [IGFBP7], which have been widely studied in common clinical settings, such as sepsis, cardiac surgery, and contrast-induced AKI. We explore the role of kidney injury biomarkers in COVID-19 and discuss what remains to be learned.

## Introduction

The coronavirus disease-2019 (COVID-19) outbreak has been declared a global pandemic, and its clinical manifestations range from mild self-limiting respiratory tract illness to severe acute respiratory distress syndrome and multiple organ failure [1]. Acute kidney injury (AKI) is a common complication of COVID-19 that is associated with higher mortality and morbidity rates [2]. The incidence rate of AKI in hospitalized patients is over 20%, and the incidence of AKI patients admitted to intensive care is over 50% [3]. When AKI occurs, dialysis rates can be as high as 30% and survival may be dramatically reduced [4]. Studies have revealed that COVID-19-associated AKI (COVID-19 AKI) has been linked to adverse outcomes, such as the development or worsening of comorbid diseases, increased mortality, and greater use of health care resources [5,6]. Therefore, early recognition of AKI in COVID-19 is crucial for reducing morbidity and mortality. However, traditional kidney functional biomarkers, such as creatinine and urine output, may be easy to misinterpret because they can be impacted by diet, body muscle mass, and sex [7]. In recent decades, several kidney injury biomarkers, including cystatin C (CysC), neutrophil gelatinase-associated lipocalin (NGAL), kidney injury molecule 1 (KIM-1), interleukin-18 (IL-18), liver-type fatty acid-binding protein (L-FABP), tissue inhibitor of metalloproteinase 2 (TlMP-2), and insulin-like growth factor binding protein 7 (IGFBP7), have been extensively studied for their value in predicting AKI in various common clinical settings, such as sepsis, cardiac surgery, and contrast-induced AKI [8–11]. The role of these kidney injury biomarkers in COVID-19, which is a complex and unique disease, has been researched [12–14]. This article first reviewed the literature on kidney injury biomarkers to summarize the performance of these biomarkers in the diagnosis or prognostication of AKI in COVID-19 and discuss what is yet to be learned.

## COVID-19-associated AKI

AKI is a prevalent complication in patients with COVID-19 [15]. The incidence of AKI in hospitalized patients is over 20%, and the incidence of AKI patients being admitted to intensive care is over 50% [3]. The dialysis rates may be as high as 30%, and the survival rate may be dramatically reduced when AKI occurs [4]. Presumably, impaired renal function and a decreased glomerular filtration rate (GFR) are likely to contribute to the development of AKI in this context. Patients who require renal replacement therapy (RRT) have a high death rate, and even those who survive AKI treated with RRT present a lack of renal recovery following discharge [16].

**Figure 1.**
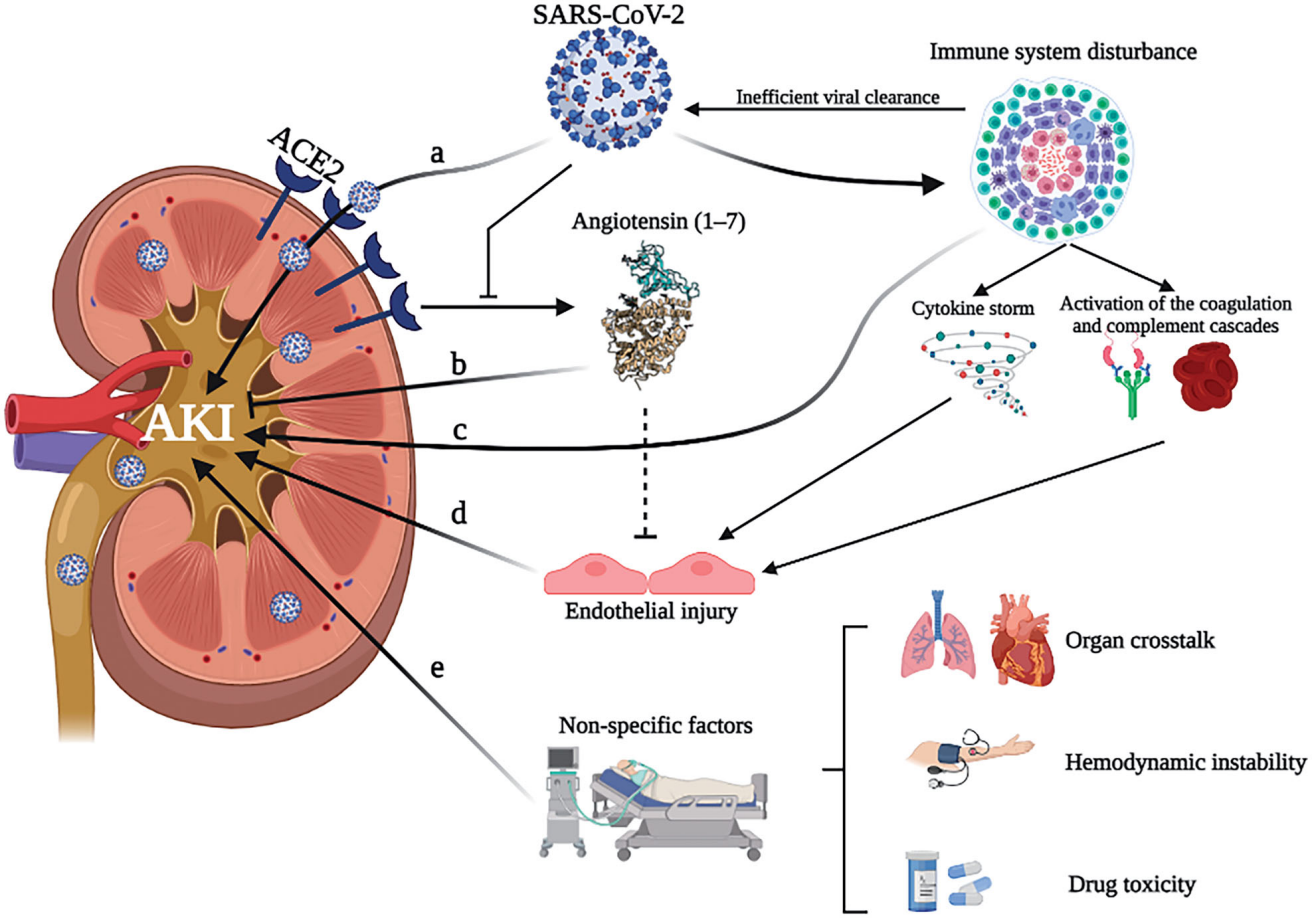
Proposed pathophysiology of COVID-19-associated acute kidney injury. (a) SARS‐CoV‐2 has been shown experimentally to infect renal tubular cells through angiotensin‐converting enzyme 2 (ACE2), which has been proposed to cause direct kidney injury. (b) Downregulation of angiotensin (1–7) caused by SARS‐CoV‐2 entry through ACE2 may aggravate acute tubular injury. (c) Following SARS-CoV-2 infection, immune system develops disturbances, including inefficient viral clearance, the enhanced release of cytokines and chemokines, activation of the coagulation and complement cascades, which may contribute to AKI. (d) Endothelial injury caused by angiotensin (1–7) inhibition and immune system disturbance may further aggravate AKI. (e) Nonspecific factors, including organ crosstalk, hemodynamic instability, and drug toxicity, will also contribute to the development of AKI.

Direct viral infection with renal tropism of the virus, overactivation of the angiotensin II pathway, dysregulated immune responses, and nonspecific factors are thought to be involved in the pathophysiology of COVID-19 AKI. Manifested by severe respiratory system attacks, COVID-19 also targets multiple organs, including the kidney [17]. Severe acute respiratory syndrome coronavirus-2 (SARS-CoV-2) has been identified and isolated from postmortem kidney tissue, and viral RNA was also detected in the kidney tissue of patients with AKI [18]. The initial impact might be direct viral infection with renal tropism of the virus mediated by activating angiotensin‐converting enzyme 2 (ACE2), which functions as a SARS-CoV-2 receptor [19]. ACE2 acts as an enzyme within the renin–angiotensin system that metabolizes angiotensin II by cleaving a terminal peptide to form angiotensin (1–7), which plays a crucial role in counteracting inflammation, vasoconstriction, and thrombosis [20,21]. SARS-CoV-2 entry, on the other hand, drastically downregulates the expression of ACE2, thereby inhibiting its protective effect, which might result in subsequent AKI triggering [20,22]. Patients with COVID-19 were reported to develop immune system disturbance, which consists of inefficient viral clearance, enhanced cytokine and chemokine release, and coagulation and complement cascade activation [23,24]. Even early cases of COVID-19 exhibited a cytokine storm, with interleukin-6 (IL‐6) playing a particularly harmful role [25,26]. IL‐6 induces renal endothelial cells to secrete proinflammatory cytokines and chemokines and promotes kidney vascular permeability and tubular and endothelial dysfunction [26,27]. Furthermore, activation of the coagulation and complement cascades may further promote the release of damage-associated molecular patterns from cells undergoing necrosis, thereby contributing to endothelial injury in COVID-19 [28–31]. The pathogenesis of COVID-19-associated AKI also involves factors that are not specific to the virus but rather aspects of a general response to critical illness or its treatment, including organ crosstalk [32–34], hemodynamic instability [35,36], and drug toxicity [37–39] (Figure 1).

## Kidney injury biomarkers

### Functional biomarkers

Current diagnostic criteria for AKI are limited by serum creatinine, which is used to calculate the estimated GFR. Muscle hypoperfusion may lead to the decreased production of creatine during infection, which blunts the increase in serum creatinine concentration and limits the early detection of AKI [40]. The reported incidence rate of AKI in COVID-19 is approximately 10% to 50% [5,41], which may be underestimated due to the shortage of this traditional kidney functional biomarker. Alternative markers for glomerular filtration have been evaluated to overcome the shortcomings of SCr in AKI settings. CysC has been identified as a potential alternative functional biomarker for AKI.

### Cystatin C

CysC is a 13-kDa endogenous cysteine proteinase inhibitor that is filtered through the glomerulus and then almost completely reabsorbed and catabolized in the proximal tubule [42]. It has been demonstrated to be superior to creatinine in the early diagnosis of AKI [42].

Yildirim et al. [43] reported that serum CysC had a high predictive value (AUC, 0.96, 95% CI: 0.90 to 1.0) for COVID-19-related AKI. The findings of the meta-analysis of 13 studies involving 2510 patients with COVID-19 indicated that higher concentrations of serum CysC were associated with higher COVID-19 severity and mortality [44]. Chen reported that elevated CysC levels were moderately predictive of disease severity in 1764 patients with COVID-19 (area under the curve [AUC]: 0.656) [45]. CysC measured at the emergency department is a highly accurate predictor of AKI and the need for RRT [46]. CysC levels have also been independently related to the risks of critical illness and mortality among patients with COVID-19 [47,48]. However, the CysC level could systematically underestimate the inulin clearance in critically ill patients. The mortality of patients with COVID-19 is related to the reduced eGFR measured by SCr rather than the reduced eGFR calculated by CysC [49], and the divergent results might be associated with inflammatory conditions and illness severity.

### Damage biomarkers

Kidney damage biomarkers reflect kidney tubule injury, which is not simply an early stage of loss of kidney function. Decreasing function and increasing damage are not as straightforward as might be assumed [50]. In most cases of AKI, a distinctive pattern may be observed in which damage proceeds to a loss of function, thus allowing for the opportunity to detect ‘subclinical’ AKI before the beginning of the loss of function. Thus, early predictive kidney damage biomarkers have great significance for clinical AKI prevention. Over the last decade, considerable progress has been made in the discovery and advancement of new kidney damage biomarkers, such as KIM-1, L-FABP, IL-18, soluble urokinase plasminogen activator receptor (suPAR), and NGAL.

### Kidney injury molecule-1

KIM-1 is a 38.7 kDa transmembrane protein with an extracellular immunoglobulin-like domain over top a long mucin-like domain [51]. KIM-1 is a biomarker for early kidney damage that has been used in a variety of clinical settings [52]. It is upregulated in the kidney proximal tubule after a wide variety of injurious influences, including ischemia, nephrotoxicants, sepsis, and immune-related injury [53,54]. The renal KIM-1 mRNA levels in patients with COVID-19 with bacterial sepsis were increased 24-fold [55]. A study published in preprint form identified KIM-1 as a receptor for SARS-CoV-2 both in the lung and kidney epithelia and indicated that it could be a potential therapeutic target in COVID-19 [56]. Few clinical studies have investigated the role of KIM-1 in patients with COVID-19. As reported by Vogel et al. [12], KIM-1 can recognize AKI at an early stage and predict a higher risk for clinical deterioration, as evidenced by ICU admissions among patients with COVID-19. The latest research showed that the urine KIM-1/creatinine ratio was associated with COVID-19-specific death [57]. However, additional research into the clinical utility of KIM-1 in patients with COVID-19 is needed.

### Liver-type fatty acid-binding protein

L-FABP is a 14 kDa protein that belongs to the large superfamily of lipid-binding proteins that can be localized predominantly in the proximal tubule [58]. L-FABP has been indicated to be a promising biomarker for a variety of kidney disorders, and it has also been shown to attenuate renal injury [59]. The L-FABP concentration was substantially lower in patients with COVID-19 than in patients with non-COVID-19 pulmonary diseases [60], while an increasing level of L-FABP was associated with adverse clinical outcomes. Tantry et al. [61] pointed out that L-FABP levels were higher in patients with clinical events, such as death, pulmonary embolism, stroke and myocardial disease, and prolonged hospitalization and mechanical ventilation requirements. Similar findings have been demonstrated by Katagiri, who claimed that L-FABP levels tended to be persistently high in severe cases, thus indicating that high levels of L-FABP were associated with severe disease in patients with COVID-19 [62]. The role of L-FABP in the early prediction of AKI in COVID-19 has yet to be fully studied. Moreover, further explorations could also focus on investigating the molecular mechanism of L-FABP in COVID-19 progression along with the effects of malabsorption and/or abnormal lipid metabolism, which may be potential therapeutic targets of COVID-19.

### Interleukin-18

IL-18 is a 22-kDa cytokine that belongs to the IL-1 superfamily, and it is activated by caspase-1 and subsequently secreted by renal tubular cells and macrophages [63]. In numerous clinical settings, the urinary level of IL-18 is expected to be an early diagnostic marker of AKI and provide prognostic information [64,65]. In response to viral infection, IL-18 is released, which induces ferritin, thus explaining the frequently observed hyperferritinemia in viral infections [66], and stimulates natural killer cell‐mediated IFN‐γ production for antiviral innate immune responses [67]. The serum concentrations of IL-18 correlate with other inflammatory markers and are linked to the severity of COVID-19 [68]. It has also been reported that serum IL-18 levels on admission are higher in patients with COVID-19 requiring mechanical ventilation and in lethal cases [69]. Schooling et al. [70] reported that IL-18 was inversely associated with any COVID-19 and very severe COVID-19. The findings of the present study shed light on the role of IL-18 in COVID-19 pathogenesis and might provide evidence for clinical trials on IL-18 antagonists for the treatment of severe patients with COVID-19. To identify the ideal time for pharmacologic IL‐18 inhibition, additional studies are required to provide a deeper understanding of the role of IL‐18 in SARS‐CoV‐2 infection.

### Soluble urokinase plasminogen activator receptor

suPAR was recently found to be a new kidney injury biomarker. The urokinase receptor system is a key regulator of the intersection among inflammation, immunity, and coagulation [71]. SuPAR is produced when membrane-bound uPAR is cleaved in response to inflammatory stimuli [72]. It has been proven to be an early biomarker in predicting AKI following cardiac surgery and in patients in the ICU [73,74]. SuPAR levels are dramatically elevated in patients with COVID-19, implying that it may be a critical mediator of COVID-19 AKI [75,76]. Azam et al. [77] indicated that suPAR levels are predictive of in-hospital AKI and the need for dialysis in patients with COVID-19. It may have a role in defense mechanisms and fibrinolysis, and low levels in severe patients may be related to poor prognosis in the early period [78]. A clinical trial involving 767 participants was carried out to investigate the role of suPAR in adult patients with COVID-19 (NCT04590794), and UPAR has been identified as a predictor of disease progression biomarkers in COVID-19 [79, 80]. Rovina and colleagues claimed that suPAR could be an early predictor of severe respiratory failure in patients with COVID-19 [75]. Moreover, Oulhaj et al. [75] indicated that suPAR has excellent prognostic utility in predicting severe complications in hospitalized patients with COVID-19. Future studies should identify the role of suPAR as a key component of the pathophysiology of AKI in COVID-19.

### Neutrophil gelatinase-associated lipocalin

The most widely investigated kidney damage biomarker of AKI is NGAL, which is a 25-kDa protein of the lipocalin family [81]. SARS-CoV-2 can infect the renal tubular epithelium directly, which may enhance the clinical value of urinary NGAL as an AKI marker among patients with COVID-19 [82]. In a retrospective study of 17 critically ill patients with COVID-19, Komaru highlighted that urinary NGAL levels were elevated in patients who went on to develop AKI during their ICU stay and that the maximum urinary NGAL value was correlated with the length of mechanical ventilation [83]. This raised the possibility that urinary NGAL could be used as an AKI biomarker in patients with COVID-19. Xu et al. [84] emphasized that urinary NGAL was strongly linked to AKI diagnosis and predicted the duration of AKI and outcomes, such as death, dialysis, shock, and longer hospital stay. He et al. [85] demonstrated that the NGAL level was an independent predictor in predicting AKI. Recently, a series of studies have shown that NGAL displayed acceptable performance for predicting AKI, the need for RRT, and death [14,46,86,87]. In addition, one clinical trial was established to study the role of NGAL and CysC in the prediction of AKI in COVID-19 infection **(**NCT04603664**)**. However, additional clinical studies should be performed in the future to determine the effect of NGAL in predicting AKI and clinical outcomes and its use in phenotyping clinical AKI in patients with COVID-19.

### Stress biomarkers

Theoretically, early stages of ‘transient AKI’ may show signs of functional decline even prior to damage. Other patterns occur as well, such as functional decline, which may start to occur alongside damage [50]. This makes damage markers difficult to employ to forecast AKI. TIMP-2 and IGFBP7 are the most extensively studied stress biomarkers.

### TIMP-2 and IGFBP7

Cell cycle arrest in the G1 phase could be a cellular mechanism to escape potential DNA damage [88]. Renal epithelial cells have been found to undergo G1 cell cycle arrest during ischemic or septic kidney injury [89]. TIMP-2 and IGFBP7 act as ‘stress biomarkers’ of two G1 cell cycle arrest urinary biomarkers, and they have been extensively validated as early kidney injury biomarkers [90,91]. Stress may occur at the cellular level prior to damage or loss of function and thus may serve as a tool for providing us with an opportunity to detect ‘subclinical’ AKI before the function starts to decline [50]. Although it may serve as an early indicator of acute kidney stress, few studies have investigated the value and clinical application of [TIMP-2] • [IGFBP7] in COVID-19. In a prospective and observational study, Husain-Syed reported that [TIMP-2] • [IGFBP7] had no effect on predicting AKI in patients with COVID-19, but higher [TIMP-2] • [IGFBP7] levels were associated with adverse clinical outcomes, including the severity of AKI, requirement of RRT, and death [92]. Gustavo et al. [86] demonstrated that elevated values of urinary [TIMP-2] • [IGFBP7] were risk factors for AKI. A clinical trial has been established to study whether TIMP-2 and IGFBP7 could identify patients with COVID-19 at risk of developing AKI early (NCT04393428), and the findings of this investigation will be made public in the future. Importantly, unlike many biomarkers, nonrenal organ failure did not result in increased [TIMP-2] • [IGFBP7] [93]. More studies should be carried out to investigate the effect of [TIMP-2] • [IGFBP7] in predicting AKI and the progress of the disease and determine its clinical usage in the phenotyping of clinical AKI in patients with COVID-19 (Table 1).

**Table 1.** Biomarkers in COVID-19-associated AKI.

Biomarkers	Publication	Design	Sample size	Clinic outcome	Sample type	AUC 95%CI	Cutoff value	Sensitivity (%) 95%CI	Specificity 95%CI	Reference
CysC	2021	Single‐center, retrospective, observational study	348	AKI	Serum	0.96 (0.90–1.0)	1.00 (mg/L)	90.0 (55.5–99.75)	88.5 (84.6–91.7)	[43]
	2020	Retrospective study	101	Mortality	Serum	0.755	0.80 (mg/L)	56.2	86.5	[48]
	2022	Single‐center, prospective, observational study	52	AKI	Serum	0.87 (0.77–0.98)	1.27 (mg/L)	70.0	96.0	[46]
				Need for RRT	Serum	0.94 (0.88–1.00)	3.22 (mg/L)	100.0	83.0	
KIM-1	2021	Prospective observational clinical trial	80	AKI	Urine	0.81	1590 ng/g UCr	87.5	65.0	[12]
				ICU	Urine	0.76	1590 ng/g UCr	79.0	64.0	
				Composite endpoint (AKI/ICU-admission/death)	Urine	0.78	1590 ng/g UCr	80.0	66.0	
	2022	2 centers, Prospective cohort study	153	Stage 3 AKI, requirement for dialysis, and death within 60 days	Urine	–	–	–	–	[14]
	2022	Prospective study	189	Death	Urine	0.749 (0.616–0.881)	1.81 (ng/mg-Cr)	77.0	70.0	[87]
L-FABP	2021	Observational study	123	Severity	Urine	–	–	–	–	[61]
	2020	Single-center retrospective study	58	Severity	Urine	0.886	9.0 μg/gCre	94.1	84.4	[62]
IL-18	2021	Observational study	58	Severity	Serum	0.90 (0.81-0.98)	576 pg/mL	78.0	77.0	[68]
suPAR	2021	Prospective study	403	Severity and Complications	Plasma	–	–	–	–	[80]
	2021	Prospective cohort study	187	Severity and mortality	Blood	0.81 (0.72-0.88)	–	82.0	65.0	[79]
	2020	Multinational observational study	352	AKI and the need for dialysis	Plasma	0.741 (0.684-0.798)	–	–	–	[77]
	2021	Observational study	120	Severity	Serum	–	–	–	–	[78]
NGAL	2021	Single-center cohort study	174	AKI and mortality	Urine	–	–	–	–	[85]
	2022	Single‐center, prospective, observational study	52	AKI	Serum	0.81 (0.68-0.95)	120(ng/L)	64.0	93.0	[46]
				Need for RRT	Serum	0.87 (0.75-1.00)	190(ng/L)	75.0	93.0	
	2022	Single‐center, prospective, longitudinal cohort study	51	AKI	Urine	0.706 (0.559-0.854)	45 (ng/mL)	54.5	76.9	[86]
	2022	2 centers, Prospective cohort study	153	Stage 3 AKI, requirement for dialysis, and death within 60 days	Urine	–	–	–	–	[14]
	2022	Prospective study	189	Death	Urine	0.750 (0.616–0.883)	118 (ng/mg-Cr)	76.0	71.0	[87]
[TIMP-2] • [IGFBP7]	2020	Single‐center, retrospective, observational study	23	AKI	Urine	–	–	–	–	[92]
	2022	Single‐center, prospective, longitudinal cohort study	51	AKI	Urine	0.682 (0.535-0.829)	0.2 (ng/mL)^2^/1000	40.0	88.4	[86]

## Conclusion

AKI caused by COVID-19 is more prevalent than initially thought and associated with morbidity and mortality. The detection of AKI with current criteria associated with the rise in serum creatinine or decrease in urine output has some limitations. Kidney injury biomarkers, such as functional biomarkers (CysC), damage biomarkers (KIM-1, L-FABP, IL-18, suPAR, and NGAL), and stress biomarkers (TIMP-2 and IGFBP7), appear to be efficient in detecting AKI as well as disease progression in patients with COVID-19. However, the majority of previous studies were single-center retrospective studies with a small number of subjects. Future well-controlled prospective studies monitoring multiple biomarkers simultaneously as well as the combination of kidney injury and damage biomarkers for the prediction of COVID-19-associated AKI should be explored. Differences in the findings for reported biomarkers in AKI with COVID-19 from other settings of clinical AKI may need to be better clarified in the future. The role of kidney injury biomarkers in the phenotyping of clinical AKI along with the potential therapeutic targets in patients with COVID-19 should also be thoroughly studied. In addition, the impact of these kidney injury biomarkers on COVID-19 variants should also be explored. Following extensive research, the field of nephrology will likely develop a deeper understanding of kidney injury biomarkers, which will aid in the clinical practice for patients with COVID-19.

## Data Availability

All data generated or analyzed during this study are included in this published article (and its supplementary information files).
